# Absence of obvious link between supra-therapeutic serum levels of β lactams and clinical toxicity in ICU patients with acute renal failure treated with intermittent hemodialysis

**DOI:** 10.1186/s13054-016-1394-2

**Published:** 2016-08-01

**Authors:** Faten May, Najouah El-Helali, Jean-François Timsit, Benoît Misset

**Affiliations:** 1 Medical Surgical ICU, Groupe Hospitalier Paris Saint Joseph, Service de Médecine Intensive et Réanimation, 185 rue Raymond Losserand, 75014 Paris, France; 2 Clinical Microbiology Unit, Saint-Joseph Hospital Network, Paris, France; 3 Department of Biostatistics, Outcomerea, Paris, France; 4 Medical ICU, Bichat hospital, Assistance Publique-Hôpitaux de Paris, Paris, France; 5 Infection, Antimicrobials, Modelling, Evolution (IAME), UMR 1137, INSERM and Paris Diderot University, Department of Biostatistics - HUPNVS. - AP-HP, UFR de Médecine, Bichat University Hospital, Paris, France; 6 Paris Descartes University, Paris, France; 7 Groupe hospitalier Paris Saint Joseph, Service de médecine intensive et réanimation, 185, rue Raymond Losserand, 75014 Paris, France

Early and adequate antibiotic therapy increases the likelihood of survival in intensive care unit (ICU) patients with sepsis [1]. Beta-lactams (BL) are the most frequently prescribed antibiotics because of their broad spectrum and low toxicity [2]. Supra-therapeutic serum levels have been suggested to be associated with clinical toxicity, mainly neurological. In the clinical settings, determining the optimal dosage is not obvious because it should take into account the distribution volume and renal elimination, both being highly variable among septic patients [3]. Consequently, serum levels of BL are unpredictable during acute renal failure (ARF) and renal replacement therapy (RRT).

To describe the prevalence of supra-therapeutic BL serum levels in septic ICU patients requiring RRT and their link with toxicity we conducted an observational retrospective cohort study. We included consecutive patients who had been sampled for a BL trough serum level assessment within 7 days of sepsis and 3 days after intermittent dialysis. Sera were sampled before the next administration in case of intermittent infusion, and at 24 h in case of continuous infusion. Table 1 shows the thresholds used and the distribution of the observed trough levels for each BL.

**Table 1:** Antibiotics assessed, thresholds used, and trough serum levels (mg/l)

	Upper therapeutic trough level^a^	Observed trough level median (interquartile range)
Piperacillin	20	77 (44–109)
Tazobactam	5	13 (6–20)
Cloxacillin	20	60 (35–103)
Amoxicillin	20	31 (19–42)
Imipenem	3	3 (1.1–4.2)
Clavulanate	0.5	2 (1.3–3.6)
Ceftazidim	20	71 (49–87)
Cefepime	10	27 (16–47)

^a^ Five times bacterial modal minimal inhibitory concentration [4, 5]

We included 108 patients, who developed 180 episodes of sepsis and were sampled 460 times. Their median Simplified Acute Physiology Score (SAPS) II was 53 (39–66) points. Seventy-four percent of the patients required vasopressors, and the overall mortality rate in the ICU was 58 %. Piperacillin (25 %), tazobactam (20 %), and cloxacillin (18 %) were the most assayed antibiotics. The distribution of the serum levels was scattered (Table 1). A supra-therapeutic serum level for at least one BL was observed in 96/108 (89 %) patients and 156/180 (86 %) septic events. A level in the highest quartile was observed for at least one BL in 54/108 (50 %) patients and 80/180 (45 %) septic events. Supra-therapeutic serum levels of piperacillin, tazobactam, and cloxacillin were observed in 66, 55, and 31, and were in the highest quartile in 33, 23, and 14 septic events, respectively. Using a univariate logistic marginal regression model to account for the correlation of successive infections in the same patient, we did not observe a statistical link between serum overdose and convulsions (*n* = 8; odds ratio 1.68 (0.15–18.9); *p* = 0.67) and mortality (*n* = 40; odds ratio 1.28 (0.38–4.33); *p* = 0.69).

Supra-therapeutic serum levels of BL antibiotics are commonly observed in our ICU patients with ARF and RRT. We could not find an association between supra-therapeutic levels and clinical toxicity.

## Abbreviations

ARF, acute renal failure; BL, beta-lactams; ICU, intensive care unit; RRT, renal replacement therapy
